# The Caregiver Support Ratio in Europe: Estimating the Future of Potentially (Un)Available Caregivers

**DOI:** 10.3390/healthcare10010011

**Published:** 2021-12-22

**Authors:** Oscar Ribeiro, Lia Araújo, Daniela Figueiredo, Constança Paúl, Laetitia Teixeira

**Affiliations:** 1 Center for Health Technology and Services Research (CINTESIS), Department of Education and Psychology, University of Aveiro, 3810-193 Aveiro, Portugal; 2 Center for Health Technology and Services Research (CINTESIS), School of Education, Polytechnic Institute of Viseu, 3504-510 Viseu, Portugal; liajaraujo@esev.ipv.pt; 3 Center for Health Technology and Services Research (CINTESIS), School of Health Sciences, University of Aveiro, 3810-193 Aveiro, Portugal; daniela.figueiredo@ua.pt; 4 Center for Health Technology and Services Research (CINTESIS), Institute of Biomedical Sciences Abel Salazar, University of Porto, 4050-313 Porto, Portugal; paul@icbas.up.pt

**Keywords:** caregivers, long-term care, oldest old, health services for the aged

## Abstract

The caregiver support ratio (CSR) is defined as the number of potential caregivers aged 45–64 years, the most common caregiving age range, for each person aged 80+, the subgroup of older adults most at risk of needing long-term services and support. This study uses data from the CENSUS HUB database and from the UN database to calculate the current (last year available: 2011) and projected (2020, 2030, 2040 and 2050) CSR for a group of European countries. Mediterranean countries, France, Belgium, and Sweden presented the lowest CSR (5:1) in 2011. The countries with the highest CSR were Slovakia (9:1) and Ireland, Poland, Cyprus, and Malta (8:1). The estimated CSR is expected to progressively decline from 6:1 (2011) to 2:1 (2050) for all countries. Although differences in the CSR exist between countries, the number of people aged 45–64 who are available to care for each person aged 80+ will decrease uniformly in the coming decades. Cross-national challenges for gerontological social policies and healthcare provision are expected due to the increasing demand for long-term care among the oldest population.

## 1. Introduction

Caregivers are defined as someone who provides continued care and assistance to a care recipient on a regular basis. The term frequently refers to family members or friends that help other people in everyday activities, whether in co-residence or not, and on a daily or weekly schedule [1]. Worldwide, family caregivers represent a significant and often invisible part in the healthcare systems, as frail older adults regularly depend on them for assistance with daily living activities, manage complex care, provide emotional support, participate in decision-making, and communicate with healthcare providers [2].

The caregiver support ratio (CSR) refers to the number of potential caregivers aged 45–64 available for each person aged 80+ [3]. In other words, it globally measures the most common caregiving age range divided by the number of older people most at risk of needing long-term service and support (LTSS).

According to the American Association of Retired Persons’ (AARP) Public Policy Institute, in 2010, more than seven potential caregivers were available for every person in the high-risk years of age 80 and over in the USA. However, by 2030, the ratio is projected to decline dramatically to 4:1, and it is also projected to decline to less than 3:1 by 2050 [3]. This suggests that by 2050, as the ratio will fall dramatically, older adults will have fewer potential caregivers, family members or friends, on whom they can rely on.

Among the OECD countries, the total share of informal carers in the population aged 50+ is 13.5%, especially women who currently account for 61.2% of carers [1]. With the expected increase in the number of older adults in the following years, the number of caregivers to be shared between care-receivers may augment in the shrinking universe of hypothetically available carers. In fact, the growing demand for care resulting from the increasing number of older adults in the population is expected to become greater in the coming years. For instance, the ratio of couples (70+) being both dependent on activities of daily living on the overall population is expected to largely increase by 2050 [4].

As of 1 January 2020, the old-age-dependency ratio in Europe was 34.8%, meaning that there were slightly fewer than three adults of working age (20–64) for every person aged 65 years or more [5]. As this ratio increases (in 2001 it was 25.9%, and by 2050 it is expected to reach 56.7%, when there will be fewer than two persons of working age to each older person), there is a decline of the workforce that is potentially available to take care of the older generations, which has already led to the increase of burden on government finances, changes to the statutory retirement age, and lower levels pf pension provision [5,6]. Adding to this, Europe also faces a rapid expansion in the number of very old people (aged 85 years or more): in 2019, the share of this age group was 2.8%, with a great majority reporting having had a long-standing illness or health problem [6].

The demographic changes that Europe faces brings several concerns for policymakers, particularly on the cost of providing adequate health and long-term care, since very old people tend to consume proportionally more social services when compared to other age groups [6] and are very likely to depend on the support of informal care, namely spouse and children, which forms the basis of all long-term care systems in Europe [7].

Forecasting the potential availability of care resources using the CSR, an easy to calculate measure of the gap in caregiving resources at the population level that has seldom been used in the European context [8], is expected to heighten the awareness of the challenges to be faced by an aging population in need of long-term services and support (LTSS). Moreover, it will allow further international comparisons with the available data for the USA, where this measure has been used to help highlight trends in family caregiving and key policy developments to support caregiving families and those for whom they care [9].

This study presents the current CSR for 28 European countries, individually and as a whole, and the projected CSR for 27 of those countries.

## 2. Materials and Methods

### Sample and Procedures

Data from the CENSUS HUB database [10] were used to estimate the CSR for 2011 (extraction date: 4 February 2021). This database was formed as the result of a major effort by the European Statistical System (ESS) to better disseminate the results of the population and housing censuses in Europe. It provides detailed data in a way that can be methodologically compared between countries.

For the CSR projections (2020, 2030, 2040 and 2050), data from the UN Population database [11] (extraction date: 4 February 2021) were used. The UN Population database is a web-based data service for the global user community that assembles a variety of statistical resources from the UN statistical system and other international agencies.

For this study, 28 countries were considered: Austria, Belgium, Bulgaria, Croatia, Cyprus, Czechia, Denmark, Estonia, Finland, France, Germany, Greece, Hungary, Ireland, Italy, Latvia, Lithuania, Luxembourg, Malta, the Netherlands, Poland, Portugal, Romania, Slovakia, Slovenia, Spain, Sweden, and the United Kingdom. Of these, one country (Cyprus) had only available data for 2011, so no projections are presented.

The caregiver support ratio (CSR) for each country in each reference year was obtained considering the ratio between the number of persons aged 45–64 and the number of person aged 80+ [3]. A linear mixed effect model was performed based on overall data (all countries and all reference years) considering CSR as the dependent variable and time as the covariate. To evaluate the trajectory of CSR across time, a linear mixed effect model was performed based on overall data (all countries and all reference years). With this purpose, CSR was considered as the dependent variable and time as the covariate. The structure of random effects comprised random intercept (country level). Estimates of coefficients and standard error (se) were reported.

All analysis were performed at R software and a significance level of 0.05 was considered.

## 3. Results

**Figure 1:**
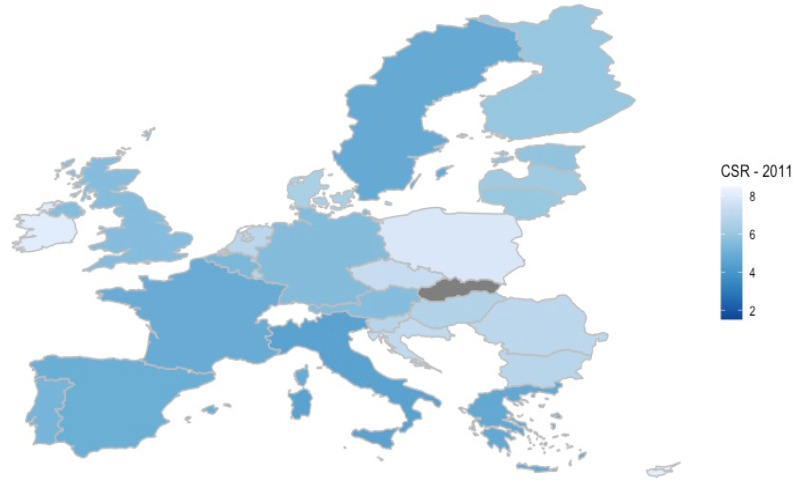
Caregiver support ratio (CSR) across Europe. (Data source: CENSUS HUB|last update: information not available|reference year: 2011|download date: 4 February 2021).

For all 28 countries, data from 2011 showed that the estimated number of potential caregivers per older person aged 80+ years old was 6:1. As seen in Figure 1, a group of Mediterranean countries (Italy, Greece, Spain and Portugal) as well as France, Belgium and Sweden presented the lowest CSR (5:1), while the countries with the highest CSR were Slovakia (9:1) and Ireland, Poland, Cyprus and Malta (8:1). Countries with a ratio of 6:1 included Austria, Estonia, Finland, Germany, Latvia, Lithuania and the United Kingdom, and countries with a ratio of 7:1 included Bulgaria, Croatia, Czech Republic, Denmark, Hungary, Luxembourg, the Netherlands, Romania, and Slovenia.

When the projected CSR for each country and for the EU as a whole was considered, a global tendency of decrease was observed. Even the countries presenting the highest number of potential caregivers in 2011 tended to approximate their ratio to the same (or close) as that projected for the EU total, which progressively decreased over the coming decades: 5 potential caregivers in 2020, 4 in 2030, 3 in 2040 and 2 in 2050. The countries showing greater decrease were Slovakia and Malta. Sweden and Italy, on the other hand, showed the lowest decrease.

**Figure 2:**
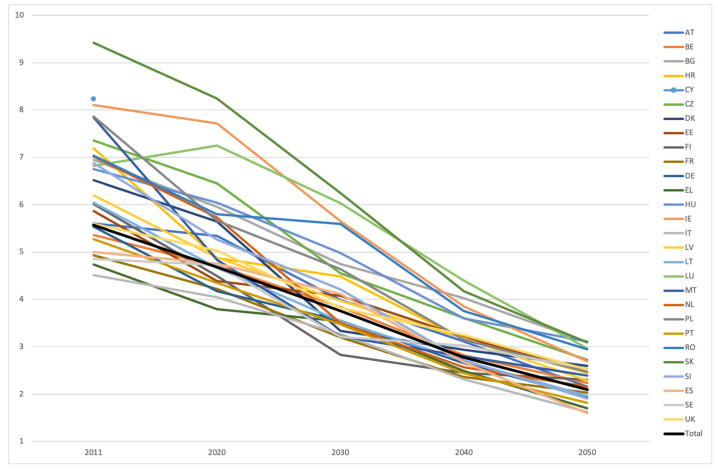
CSR across EU countries (Data source: CENSUS HUB (2011) and UNdata [2020–2050]|last update: information not available (2011) and 17 June 2019 (2020–2050)|reference years: 2011, 2020, 2030, 2040 and 2050|download date: 4 February 2021).

Figure 2 illustrates the global tendency observed throughout the considered countries, and Table 1 presents specific data for each country and in total.

Overall, 10 countries were expected to have 3 adults aged 45–64 per each older person aged 80+ in 2050; for the other 17 countries, this ratio was 2:1, with Spain presenting the lowest value (1.59). Through performing a linear mixed effect model and considering CSR as the dependent variable and time as the covariate, the significant decrease of CSR was, on average, 1.07 (se = 0.16) from 2011–2020, 2.23 (se = 0.14) from 2011–2030, 3.28 (se = 0.14) from 2011–2040 and 4.00 (se = 0.14) from 2011–2050.

## 4. Discussion

Following the original report published by the AARP Public Policy Institute (USA), CSR was used to estimate the availability of potential caregivers in the EU, both currently and over the upcoming decades. The observed demographic trends highlight that, overall, the CSR is projected to shrink from six potential caregivers per older person today (as in 2011) to just four per person in 2030. The analysis of the possible future availability of caregivers depends on many processes of social change that influence its supply (e.g., whether the family caregiver is employed and working at paying job, the declining birth rate with families having fewer children, the extent of inter-generational living, the proportion of women in the workforce, the availability of paid direct care workers) and are likely to significantly differ across the countries examined. However, due to the change in co-residence of older people and the availability of and solutions for conjoint work with formal services, among others [12], policy decisions regarding LTSS must account for an expected transversal care gap in the coming decades, as the CSR may further decline to 3:1 and 2:1 in 2040 and 2050, respectively. In other words, throughout Europe there will be increasing pressures on fewer individuals within families to care for older adults in the high-risk years of eighty and older.

Although the CSR does not have a unique distribution by geography, welfare systems [13] or elder care country-clusters and care regimes (family-based, standard care-mix, public-Nordic, transition, Baltic) [14], what seems clear is the fact that four Mediterranean countries (Spain, Portugal, Greece, and Italy) presented the lower values of CSR practically in all time periods considered in this analysis. The fact that these countries face problems in terms of poverty and social exclusion [15], and are among the European Member states where the old-age dependency ratio is projected to reach a level of at least 60 percent by 2050 (66.5% in Italy, 68.1% in Greece, and 68.8% in Portugal), presenting the highest share of very old people already in 2019 [6], makes it particularly urgent to acknowledge their expected challenges in facing greater investment needs in family caregiver support and greater demand for paid LTSS. In fact, the provision of long-term care to frail older adults is a pressing policy issue, and it has been reported a strong North-South gradient in the utilization of informal care across Europe, with countries like Italy and Spain presenting the greater percent of all care hours (up to 81), when compared to Northern countries (Belgium, Denmark, the Netherlands, and Sweden) and Middle countries (Austria, France and Germany), where of all care hours, informal care accounts for 22% and 43%, respectively [16].

The number of European older adults aged 80+ will exponentially increase in the coming years [17]. Particularly, the population aged 90+ is at greater risk of physical dependency due to long term-conditions, disabilities, or frailty, and thus very likely to need LTSS, which includes day-to-day help (e.g., personal care, help with medication intake, help with housekeeping, transportation, paying bills) that can be provided in the home, in assisted living, in nursing facilities, and in integrated settings. But LTSS also include supportive services that are provided by family members and other unpaid caregivers. Therefore, the next two or three decades of elder care may tax the health and economic wellbeing of the next generation of family caregivers [18] and certainly be a societal and a healthcare challenge. In this sense, not only accessible and good (public) long-term care services for the person in need of care will have to be assured, but also a greater investment in directly supporting family and friends in their caregiving roles will have to be guaranteed. The range of possible supportive measures is wide and includes recognition of legal rights, information, advice and emotional support, counselling, financial support (e.g., income compensation, expenditure compensation, time compensation), training and education, respite care services, peer support, advocacy, health-related support (e.g., health check-ups), and technological/ICT solutions for adjusted housing, adjusted living and adjusted care [14]. Although most countries face similar challenges regarding the provision of care to older adults, particularly the sustainability of informal care, the policy responses vary greatly throughout Europe. A key transversal point, however, relies in recognizing the informal carers’ needs as early as possible, as well as offering supportive services on an ongoing basis [7]. Additionally, long-term-care and mainstream care cannot continue to be disconnected: an integrated system must be considered. One where not only long-term care is recognized as a public good both societally and politically, but that also ensures an acceptable level of well-being for the dependent older people and for those who care for them [19], i.e., a coordinated care delivery in health care and LTSS that focuses on person- and family-centered care as a standard practice.

The longevity phenomenon also calls attention to the worldwide decline in the oldest old support ratio (defined as the number of people aged 50–74 divided by the number of people aged 85+), which is also expected to decline from 32.0 in 2005 to 12.5 in 2050 [20]. Within the scope of an expected greater demand for care due to the increasing number of near-centenarians and centenarians in Europe (the numbers of people aged 100 years or more is projected to be close to half a million in the EU-27 by 2050 [6]), meeting the compound caregiving needs of this population who may present extended years of disability and are very likely to live at home [21] is an additional topic of concern in the years to come [22], especially if caregivers are very old themselves and face complex care constellations [23]. A recent report on the emotional well-being of older carers from the UK exposed the multitude of strains created by caregiving in this particular group (carers over the age of 65), providing evidence from research that two thirds have long term problems or a disability themselves, and that, overall, long term caregiving was associated with declines in quality of life and life satisfaction, with an increased risk of depression for both males and females, and with deleterious socio-emotional effects after giving up caregiving, suggesting the need for services that provide appropriate support at all stages of the caregiving cycle in this stage of life [24]; moreover, the same report highlighted that the number of carers over 65 rose 35% since 2001.

A recent study that aimed to estimate the prevalence of older informal caregivers (aged 50+) in Europe based upon three European surveys (EHIS—European Health Interview Survey; EQLS—European Quality of Life Survey; and SHARE—Study on Health and Ageing in Europe) showed rates that ranged between about 13% in Portugal and Spain, and more than 22% in Luxembourg, Belgium, and Denmark [25]. Although globally declining in older age groups, particularly from the late fifties, and presenting great variability between countries (in every age group, as well as within each country and across the three surveys), the study also showed that the share of informal caregivers aged 65–69 years old, 70–74 years old, and aged 75+, reached up to 38.30, 31.71 and 26.91, respectively. As these shares are quite expressive, and as the oldest age groups are likely to become one of the most rapidly growing segments of the caregiver population, paying them particular attention in research in the future is of crucial importance [26]. Moreover, as they may be simultaneously caregivers and care-receivers, accounting for specificities in the healthcare provision to these caregiving dyads may also be needed.

This study findings also call to consider the availability of formal caregivers in the upcoming years. Given the fact that most of the long-term care workers are middle-aged [27], the lower CSR expected for the next decades can (and will) lead to serious shortcomings in the provision of care for older persons, which in the case of LTC have health problems that deserve even more support. Along with the demographic changes, the OECD also calls attention to the problems of this sector, such as poor working conditions, which may aggravate even more the potential supply of labor in LTC.

This eminently descriptive study takes up to discussion the issue of the estimated prevalence of caregivers throughout Europe but has several limitations that should be conveniently addressed when interpreting the findings. Although it considers reliable, validated, and trustful statistics as the census data (currently only available for 2011), the presented projections are subject to estimative errors and population changes, as it is the case of the COVID-19 pandemic which will certainly have influence in the CSR in the coming years. In fact, after 2020 it is expected a lost in life expectancy, largely attributable to increased mortality above those aged 60+ years linked to official COVID-19 deaths [28], and this will probably impact the ratio of older adults in need of support vs. the number of potential caregivers of younger ages. A second important limitation is inherent to the concept of CSR itself, as it assumes that all the population within the age range of 45–64 has the capacity to provide care, which does not correspond to reality; therefore, cautious interpretations are required. Lastly, it will be important to calculate the CSR in the near future considering up to date statistics from the census in 2021—this will provide a more realistic picture of the observed CSR pattern and projections.

## 5. Conclusions and Future Direction

As an elementary measurement of the potential availability of caregivers, the CSR considers the number of people in the population that is available to arrange, coordinate and provide LTSS. The estimated decline in the CSR throughout Europe for the next decades signals an important squeeze in the number of potential family caregivers available to care for an 80-plus cohort that is rapidly outpacing those aged 45–64. Given the strong reliance on informal care throughout Europe, namely in Mediterranean countries, understanding the current and future availability of informal care in each country is particularly important for facing the challenges of integrated LTC systems. The provision and financing of LTC by the public sector is expected to be under strong pressure in the future due to reduced availability (and/or propensity) of potential informal carers to provide care. The lack of informal carers may force dependents to move to institutional care or lead to an increase in demand for home care. Anticipating future trends in LTC spending is essential to devise appropriate policies and ensure that good quality and accessible services are provided. Meeting the informal caregivers’ needs is currently a socio-political topic of concern in Europe, and within the scope of an expected greater demand for care due to the increasing number of frail oldest-old individuals like (near) centenarians, it would be of relevance to further estimate the oldest old caregiver support ratio, considering this age group and its potentially associated older generation of care providers (65+). The repercussions of the future CSR and the role of (un)paid care providers and facilities are therefore incontrovertible topics of reflection within healthcare provision and social policy responses to an aging population.

## Figures and Tables

**Table 1:** Caregiver support ratio (CSR) by European country, 2011–2050.

Country	Caregiver Support Ratio, by Year				
	2011	2020	2030	2040	2050
Austria	5.60	5.34	3.99	3.11	2.16
Belgium	5.36	4.71	3.84	2.82	2.24
Bulgaria	6.95	5.95	4.75	4.03	3.10
Croatia	7.19	4.87	4.48	3.15	2.45
Cyprus	8.24	-	-	-	-
Czech Republic	7.36	6.45	4.55	3.61	2.72
Denmark	6.52	5.64	3.34	2.94	2.60
Estonia	5.87	4.39	4.08	3.21	2.47
Finland	6.01	4.49	2.83	2.45	2.30
France	4.93	4.22	3.20	2.36	2.03
Germany	5.54	4.17	3.55	2.65	1.94
Greece	4.74	3.79	3.54	2.49	1.70
Hungary	6.75	6.04	4.98	3.60	3.11
Ireland	8.11	7.72	5.65	3.85	2.69
Italy	4.51	4.05	3.27	2.31	1.62
Latvia	6.20	4.88	3.97	3.18	2.28
Lithuania	6.04	4.67	3.54	2.70	1.96
Luxembourg	6.82	7.25	6.03	4.40	2.97
Malta	7.84	4.83	3.22	2.80	2.39
Netherlands	7.02	5.73	3.50	2.57	2.16
Poland	7.86	5.66	4.64	3.15	2.47
Portugal	5.27	4.35	3.47	2.44	1.82
Romania	7.03	5.80	5.59	3.75	2.95
Slovakia	9.42	8.24	6.25	4.17	3.09
Slovenia	6.88	5.26	4.21	2.71	1.90
Spain	5.00	4.76	4.11	2.67	1.59
Sweden	4.85	4.73	3.21	3.02	2.54
United Kingdom	5.59	5.03	3.82	3.25	2.52
Total	5.57	4.68	3.76	2.78	2.09

(Data source: CENSUS HUB (2011) and UNdata (2020–2050)|last update: information not available (2011) and 17 June 2019 (2020–2050)|reference years: 2011, 2020, 2030, 2040 and 2050|download date: 4 February 2021).

## Data Availability

See References [10,11].
